# iSupport for Dementia: an analysis of clinical trial records

**DOI:** 10.1192/j.eurpsy.2023.987

**Published:** 2023-07-19

**Authors:** S. C. I. Pavarini, A. C. Ottaviani, D. Monteiro, C. Campos, L. Corrêa, L. Alves, L. Rocha, G. Barbosa, A. Cardoso, L. Maciel, E. Barham, D. Oliveira, K. Cruz, F. Orlandi, A. G. Gratão

**Affiliations:** 1 Departament of Gerontology; 2 Postgraduate Programme in Psychology; 3 Postgraduate Programme in Nursing; 4 Department of Psychology, Universidade Federal de São Carlos, São Carlos; 5 Department of Psychiatry, Universidade Federal de São Paulo, São Paulo; 6 Department of Nursing, Universidade de Brasília, Brasília, Brazil

## Abstract

**Introduction:**

Dementia has a significant psychological and emotional impact on families, especially for caregivers of people living with dementia. In this perspective, the World Health Organization has developed iSupport for Dementia, an online training and skills program to prevent and/or reduce mental health problems associated with the provision of care and improve the quality of life of caregivers. It is being translated and adapted in different countries and as of August 2022, 31 adaptations using 27 different languages were in progress. However, the availability of the program should only be carried out after evaluating its effects on caregivers’ mental health outcomes (such as burden, depressive and anxious symptoms, quality of life, among others).

**Objectives:**

To analyze randomized clinical trial protocols to assess the effects of the iSupport program in different countries.

**Methods:**

This is a data survey carried out in October 2022 on clinical trial registry platforms Clinical Trials, The Brazilian Registry of Clinical Trials, Cochrane Central Register of Controlled Trials, Netherlands Trial Register and Australian New Zealand Clinical Trials Registry by two independent researchers. Descriptive analyzis were performed for sample size, primary outcomes, secondary outcomes and intervention design.

**Results:**

Seven clinical trial registries were identified, conducted in Australia/China, Brazil, Great Britain, the Netherlands, India, Japan and Portugal, published in English, from 2018 to 2022. The sample size ranged from 184 to 390 participants. Regarding the primary outcomes linked to the effect of using iSupport, five countries will analyze burden, anxiety and depression. Only in Australia/China and the Netherlands, the primary outcome will be quality of life and stress, respectively. Secondary outcomes vary between studies, with measures of quality of life (n=6), self-efficacy (n=4), program usability (n=4), cognition and problematic behaviors (n=3), attitudes (n=3), quality of support (n=3), positive aspects of care (n=2), knowledge, competence, resilience and informal costs of care (n=1). Most studies will carry out assessments at baseline, 3 and 6 months after the intervention, with the exception of Japan that will perform at baseline and at 1 and 3 months after the intervention and 6 months.

**Conclusions:**

Analysis of the effectiveness of iSupport is one of the World Health Organization guidelines for countries that are culturally adapting this program. Brazil is the only country in Latin America with a clinical trial registration so far. Burden, anxiety and depression are outcomes considered by most countries. The results could provide evidence to strengthen and expand the possibilities for collaboration between researchers, as internet-based interventions have shown promising results on the mental health and well-being.

**Disclosure of Interest:**

None Declared

